# Short-term effectiveness of baricitinib in children with refractory and/or severe juvenile dermatomyositis

**DOI:** 10.3389/fped.2022.962585

**Published:** 2022-09-20

**Authors:** Zhaoling Wang, Qi Zheng, Wenjie Xuan, Xisheng Xu, Meiping Lu, Jianqiang Wu, Lixia Zou, Yiping Xu, Xuefeng Xu

**Affiliations:** ^1^Department of Rheumatology Immunology and Allergy, Children’s Hospital of Zhejiang University School of Medicine, National Clinical Research Center for Child Health, Hangzhou, China; ^2^Department of Pediatric, Shaoxing People’s Hospital, Shaoxing, China

**Keywords:** baricitinib, juvenile dermatomyositis, refractory, severe, treatment

## Abstract

**Objective:**

To determine the short-term effectiveness safety of baricitinib in children with refractory and/or severe juvenile dermatomyositis (rsJDM) in a real-world setting.

**Methods:**

This was a single-center retrospective study, including 20 children with rsJDM. They were all treated using baricitinib combined with steroids and other immunosuppressive agents. The childhood myositis assessment scale (CMAS) and PRINTO remission criteria were used to evaluate the disease severity and treatment outcome at 0, 4, 12, and 24 weeks after initiation of baricitinib.

**Results:**

The skin rash improved in 95% of patients (19/20) at week 24, with a significant decrease of skin-DAS at weeks 12 (6.0 vs. 2.0, *p* < 0.05] and week 24 [6.0 vs. 1.0, *p* < 0.05) by median statistics. The CMAS score increased significantly at week 12 (41.0 [29.0, 44.0] vs. 46.0 [42.0, 52.0], *p* < 0.05) and week 24 (41.0 [29.0, 44.0] vs. 50.0 [45.0, 52.0], *p* < 0.05), as did the manual muscle testing (MMT)-8 score at week 24 (73.0 [610, 76.0] vs. 79.0 [77.0, 80.0], *p* < 0.05). At 24 weeks, the complete response (CR) and partial response (PR) were achieved in 75% (15/20) and 15% (3/20), respectively. The dose of corticosteroids (CS) decreased by 37% from the baseline (0.53 [0.42, 1.00] mg/kg) to week 12 (0.33 [0.18, 0.40] mg/kg) (*p* < 0.05), and by 49% at week 24 (*p* < 0.05). No serious side effects were observed.

**Conclusion:**

Baricitinib combined with traditional immunosuppressants treatment was efficacious in rsJDM. Add-on therapy of baricitinib was helpful for tapering CS dose. No serious side effects were observed in this study.

## Introduction

Juvenile dermatomyositis (JDM) is an autoimmune disease characterized by inflammation of both the skin and muscles. The typical skin signs of JDM are Gottron papules, heliotrope rashes, and nailfold capillary changes (1). Other organs such as the lungs, the heart, and the gastrointestinal tract can also be involved, which would result in progression to severe JDM (sJDM). Although the prognosis of JDM has been remarkably improved by traditional drugs including corticosteroids (CS) and other immunosuppressive agents, 20% of cases still develop refractory JDM (rJDM) or even become unresponsive to the above therapy (2). Other therapies such as immune support and biologic therapies have yielded some promising results, but disease relapse, difficulty in tapering CS dose, and adverse effects (AEs) due to long-term CS use are still hindrances in the treatment course (3–6). Recent studies have found that interferons (IFNs) play a key role in dermatomyositis (DM) pathogenesis (7, 8). Elevated signatures of types I and II IFNs have been found in the tissues and cells of patients with DM (9–11), with signal transduction reliant on the Janus kinase (JAK)-signal transducers and activators of the transcription pathway. Given the presence of IFN dysregulation in the JDM, IFN-targeting therapies have been attempted. Janus kinase inhibitors such as tofacitinib (JAK1/3 inhibitor), ruxolitinib (JAK1/JAK2 inhibitor), and baricitinib (JAK1/2 inhibitor) were studied in patients with JDM (12–19). However, there are insufficient data about the effectiveness and safety of baricitinib in JDM, with only eight cases reported worldwide: three by Voyer et al. (15), four by Kim et al. (17), and one by Papadopoulou et al. (18). Here, we report on 20 cases with refractory and/or severe juvenile DM (rsJDM) that received baricitinib combined with tradition drugs in a real-world setting to evaluate its effectiveness and safety in a real-world setting.

## Methods

### Patients

We retrospectively analyzed 20 cases with rsJDM who received baricitinib for 12–24 weeks combined with CS and immunosuppressive agents between January 2019 and January 2022. The diagnosis of JDM was based on the Bohan and Peter classification (20). The study was approved by the Ethics Committee of Children’s Hospital of the Zhejiang University School of Medicine (IRB approval no. 2019-IRB-154). In total, twenty-two patients treated with baricitinib were included, and two patients were excluded. In total, one case was excluded due to incomplete clinical data, and the other was due to the loss of follow-up. Of the remaining patients, the median [interquartile range, IQR] value of the baricitinib dose was 0.05 [0.04, 0.09] mg/kg/day. The median time [IQR] from diagnostic to baricitinib onset was 16.5 [8.8, 35.3] months. Baricitinib was administered to 17 cases for 24 weeks, and to the other 3 for 12 weeks. The subjects were mean aged 7.8 ± 4.0 y, and their women-to-men ratio was 3:1. Clinical and laboratory data were analyzed at the baseline (week 0) and at weeks 4, 12, and 24 after baricitinib administration.

The inclusion criteria were: (i) diagnosis of JDM according to the Bohan and Peter classification and meeting the diagnostic criteria for rJDM and/or sJDM, (ii) all the patients with JDM received baricitinib (at least 12 weeks), (iii) follow-up at least 24 weeks after the initiation of baricitinib.

The exclusion criteria were: (i) myositis overlapping with other autoimmune diseases, (ii) evidence of any other acute or chronic infectious disease, (iii) history of malignancy in any organ system, (iv) incomplete clinical and laboratory data, and (v) history of hypersensitivity to baricitinib.

### Study definitions

Patients who met the diagnostic criteria for rJDM and/or sJDM, rJDM were defined by inadequate response to glucocorticoids and at least one other first-line immunosuppressive agent (e.g., azathioprine, methotrexate, mycophenolate mofetil, cyclosporine, tacrolimus, cyclophosphamide, leflunomide, or IVIg) (6). The sJDM was referring to young-onset (age < 1 year), severe muscle involvement (CMAS score < 15 or MMT-8 score < 30) or severe skin involvement (ulcerative skin disease), or involvement of other organs and systems, such as gastrointestinal and cardiac manifestations, interstitial lung disease, development of calcinosis, or need intensive care unit management (21). The ILD was defined based on the findings of the chest radiography and the chest CT scans that were evaluated by radiologists (22, 23).

### Effectiveness evaluation of baricitinib in juvenile dermatomyositis

Skin disease activity was assessed using the skin disease activity score (skin-DAS, which ranges from 0 to 9) (24). Muscle strength was assessed using the childhood myositis assessment scale (CMAS, score range 0–52), and the Manual Muscle Testing-8 scale (MMT-8, score range 0–80). A complete response (CR) was defined by both the Paediatric Rheumatology International Trials Organisation (PRINTO) remission criteria and inactive skin-DAS (15). The PRINTO remission comprised meeting at least three of the four following criteria: creatine kinase ≤ 150 U/L, CMAS score ≥ 48, MMT-8 score ≥ 78, and physicians’ global activity of ≤2 cm on a visual analog scale ranging from 1 to 10 cm (20). Inactive skin-DAS was defined by a score ≤ 1/9 without cutaneous ulcerations or erythema. A partial response (PR) was defined as improvements in CMAS/MMT-8 score and skin-DAS, allowing CS dosage tapering of at least 50% from the initial dosage, without combining with a new immunosuppressive agent (25).

### Detection of myositis antibodies

The anti-myositis antibody spectrum Ig-G detection kit (Lot No.: DL1530-1601-4G) was applied to detect myositis antibody (Omon Company of Germany). The detection kit contains 15 anti-myositis antibodies (Mi-2α, Mi-2β, TIF1γ, MDA5, NXP2, SAE1, Ku, PMScl100, PM-Scl75, Jo-1, SRP, PL-7, PL-12, EJ, and OJ, Ro-52). Test results on the EUROBlotMaster II. Staining intensity > 15 is positive (EURO Line Scan).

### Statistical analysis

Statistical analyses were performed using IBM SPSS Statistics (version 25) and GraphPad Prism (version 8.0.1) software. The data were analyzed using the Friedman test. The continuous variables were presented as medians with interquartile ranges or means with standard deviations. The categorical variables were presented as the number of patients and percentages. A probability value of *p* < 0.05 was considered significant.

## Results

### Demographic, clinical, and laboratory data and drug combinations at baseline

All patients (*n* = 20) had skin involvement, namely, recurrent or refractory skin rashes (*n* = 19, 95%) and calcinosis (*n* = 3). The median skin-DAS was 6.0 [4.0, 7.3]. In total, seven patients had muscle involvement. The median CMAS score was 41.0 [29.0, 44.0], while the median MMT-8 score was 73.0 [61.0, 76.0] (Table 1, Supplementary material). The median creatine kinase was 93.5 [52.5, 178.0] U/L. In total, five patients had interstitial lung disease (ILD), and one patient who received baricitinib at the initial onset of JDM (P9) had complicated macrophage activation syndrome (MAS) (Table 2). At the baseline, patients received CS (*n* = 20), intravenous methylprednisolone (IVMP, *n* = 1), intravenous immune globulin (IVIG, *n* = 9), methotrexate (MTX, *n* = 15), hydroxychloroquine (HCQ, *n* = 2), tacrolimus (*n* = 2), mycophenolate mofetil (MMF, *n* = 1), cyclosporine A (CsA, *n* = 1), thalidomide (*n* = 1), infliximab (*n* = 1), and tocilizumab (*n* = 1).

**TABLE 1:** Effectivenessand daily dose of corticosteroids (CS) in patients with baricitinib treatment.

Score	Week 0	Week 4	Week 12	Week 24
Total-DAS	6.8 (5.5, 9.3)	5.0 (3.0, 6.4)	2.5 (2.0, 3.0)	1.0 (0, 2.4)
Skin-DAS	6.0 (4.0, 7.3)	4.5 (2.0, 5.0)	2.0 (0, 3.0)^a^	0 (0, 1.0)^b/c^
CMAS	41.0 (29.0, 44.0)	42.0 (38.0, 47.0)	46.0 (42.0, 52.0)^d^	50.0 (45.0, 52.0)^e^
MMT-8	73.0 (61.0, 76.0)	75.0 (71.0, 77.0)	78.0 (72.0, 80.0)	79.0 (77.0, 80.0)^f^
PGA	5.0 (5.0, 7.0)	4.0 (3.0, 5.0)	3.0 (0.3, 3.8)	0 (0, 2.8)
PaGA	6.0 (5.0, 6.0)	3.0 (0, 5.0)	0 (0, 4.0)	0 (0, 0.8)
CK	93.5 (52.5, 178.0)	100.0 (54.0, 128.0)	84.0 (58.3, 102.5)	96.0 (70.3, 130.8)
LDH	258.0 (231.3, 413.3)	275.5 (244.3, 397.5)	265.5 (220.5, 289.3)	256.5 (218.8, 290.8)
CS dosage	0.53 (0.42, 1.00)	0.40 (0.23, 0.65)	0.33 (0.18, 0.40)^g^	0.27 (0.17, 0.37)^h/i^

^a^: week 12 vs. week 0, *p* < 0.05; ^b^: week 24 vs. week 4, *p* < 0.05; ^c^: week 24 vs. week 12, *p* < 0.001; ^d^: week 12 vs. week 0, *p* < 0.023; ^e^: week 24 vs. week 0, *p* < 0.006; ^f^: week 24 vs. week 0, *p* < 0.011; ^g^: week 12 vs. week 0, *p* < 0.05; ^h^: week 24 vs. week 0, *p* < 0.05; ^i^: week 24 vs. week 4, *p* < 0.007; DAS: disease activity score (*n* = 20); CMAS: childhood myositis assessment scale (*n* = 7); MMT-8: manual muscle testing-8 (*n* = 7); PGA: physicians global activity (*n* = 20); PaGA: parent global activity (*n* = 20); CK: Creatine kinase (*n* = 20), normal range 39.0∼308.0 U/L; LDH: Lactate Dehydrogenase (*n* = 20), normal range 110.0∼295.0 U/L; CS: corticosteroid (*n* = 18).

**TABLE 2:** General informations of patients receiving baricitinib therapy and outcomes (*n* = 20).

Patient	Age at diagnosis years/sex	Duration of diagnostic to baracitinib onset (Month)	Clinical characteristics before baricitinib treatment (week 0) skin-DAS, CMAS-14,MMT-8	Main indication for baracitinib treatment	Muscle biopsy features	MSAs/ MAAs	Treatment/ duration (month)	Dose of baricitinib (mg/kg/d)/ (mg/ frequency)	Outcome (week)
1	10/F	17.5	8/9,52/52,80/80	Refractory skin rash	Endomysial infiltration of mononuclear cells surrounding, but not invading, myofibers	Negative	CS^a^ + MTX^a^ + HCQ^b^ CS^a^ + MTX^a^ + Bari/6	0.05 2 mg, qd	PR/4,CR/12 CR/24
2	11/M	24.7	7/9,44/52,77/80, calcinosis	Refractory skin and muscle involvement	Perifascicular atrophy	Negative	CS^d^ + MTX^d^ + IVIG^c^ + INF (Bimonthly) CS^d^ + MTX^d^ + IVIG^c^ + INF (Bimonthly) + Bari*/3 CS^d^ + MTX^d^ + IVIG^c^ + INF (Bimonthly) + Thalidomide^e^/3	0.05 2 mg, qd	NR
3	8/F	15.5	8/9,15/52,60/80, calcinosis	Calcinosis	Perifascicular atrophy	MDA5/Ro52	CS^a^ + LEF^b^ + MTX^d^ + IFX^b^ + IVIG^c^ CS^a^ + MTX^d^ + IVIG^c^ + Bari/6	0.05 2 mg, qd	PR/24
4	6/F	3.3	7/9,52/52,80/80,ILD	Refractory skin rash	N/A	MDA5/Ro2	CS^a^ + MTX^b^ + CsA^b^ + HCQ^d^ + IVIG^c^ CS^a^ + HCQ^d^ + IVIG^c^ + Bari/6	0.05 1 mg, qd	PR/12,CR/24
5	10/M	41.9	3/9,47/52,75/80,ILD	Complications (ILD) and refractory skin rash	Perimysial and/or perivascular infiltration of mononuclear cells	NXP2	CS^a^ + MTX^b^ + HCQ^e^ + IVIG^d^ CS^a^ + HCQ^e^ + IVIG^d^ + Bari*/6	0.04 2 mg, qd	NR
6	11/M	10.9	8/9,29/52,61/80	Refractory skin and muscle involvement	Focal atrophy, perimysial and/or perivascular infiltration of mononuclear cells	NXP2	IVMP (once) CS^a^ + MTX^d^ + HCQ^d^ + IVIG^d^ CS^a^ + MTX^d^ + HCQ^d^ + IVIG^d^ + Bari/6	0.05 2 mg, qd	PR/4,PR/12 CR/24
7	2/F	71.2	5/9,50/52,74/80	Refractory skin rash	Perimysial infiltration of mononuclear cells	NXP2	IVMP (once) CS^c^ + MTX^d^ CS^c^ + MTX^d^ + Bari/6	0.04 1 mg, qd	PR/4,CR/12 CR/24
8	3/M	7.8	5/9,52/52,80/80	Refractory skin rash	Focal atrophy, perimysial and/or perivascular infiltration of mononuclear cells	TIF1γ	CS^a^ + MTX^d^ + IVIG^c^ CS^a^ + MTX^d^ + IVIG^c^ + Bari/6	0.05 1 mg, qd	CR/4
9	10/F	0	8/9,41/52,73/80,ILD, MAS	Newly-onset severe case	Perivascular infiltration of mononuclear cells	PL-12	IVMP (once) CS + MTX^d^ + CsA^c^ + Tocilizumab^c^ + IVIG^c^ CS + MTX^d^ + CsA^c^ + Tocilizumab^c^ + IVIG^c^ + Bari/6	0.04 1.5 mg, qd	PR/4,PR/12 CR/24
10	1/F	29.6	6/9,52/52,80/80	Refractory skin rash	N/A	SRP/PM-SCl75	IVMP (once) CS^a^ + MTX^a^ + IVIG^d^ CS^a^ + MTX^a^ + IVIG^d^ + Bari/6	0.16 2 mg, qd	PR/12, CR/24
11	8/F	4.2	3/9,52/52,80/80, CADM	Refractory skin rash	N/A	TIF1γ/Ro52	IVMP (once) CS^a^ + MTX^a^ + HCQ^c^ CS^a^ + MTX^a^ + HCQ + Bari/6	0.07 2 mg, qd	PR/4,CR/12 CR/24
12	6/F	59	6/9,50/52,78/80, calcinosis	Skin ulcerations	N/A	NXP2/PM-SCl75	CS^d^ + CsA^b^ + CTX^b^ + rhTNFR:Fcb + HCQ^c^ + IVIG^c^ CS^d^ + HCQ^c^ + IVIG^c^ + Bari/6	0.12 2 mg, bid	PR/24
13	10/F	15.2	3/9,49/52,78/80	Refractory skin rash	Perivascular infiltration of mononuclear cells	Negative	IVMP (twice) CS^a^ + MTX^d^ + Tacrolimus^d^ + IVIG^c^ CS^a^ + MTX^d^ + Tacrolimus^d^ + IVIG^c^ + Bari/6	0.04 2 mg, qm + 1.5,qn	PR/4,PR/12 CR/24
14	14/F	10.5	6/9,52/52,80/80,ILD	Refractory skin rash and complications (ILD)	Focal atrophy, perimysial and/or perivascular infiltration of mononuclear cells	PL-7/Ro52	CS^d^ + CTX^b^ + MMF^d^ + IVIG^c^ CS^d^ + MMF^d^ + IVIG^c^ + Bari/6	0.04 2 mg, bid	PR/4,PR/12 CR/24
15	9/M	34.6	5/9,52/52,80/80	Refractory skin rash	N/A	NXP2	IVMP (once) CS^d^ + MTX^b^ + Tacrolimus^c^ + IFX^b^ + IVIG^c^ CS^d^ + Tacrolimus^c^ + IVIG^c^ + Bari/6	0.09 2 mg, bid	PR/12, CR/24
16	12/M	3	5/9,52/52,80/80	Refractory skin rash	N/A	PL-7/Ro52	CS^a^ + MTX^d^ + IVIG^d^ CS^a^ + MTX^d^ + IVIG^d^ + Bari/6	0.04 2 mg, qm + 1 mg, qn	PR/4,PR/12 CR/24
17	14/F	14.5	3/9,52/52,78/80,ILD	Refractory skin rash and complications (ILD)	Degeneration of muscle fibers, perimysial infiltration of mononuclear cells	MDA5	CS^a^ + MTX^d^ + IVIG^c^ + Tacrolimus^d^ + HCQ^c^ + CTX^d^ CS^a^ + MTX^d^ + Tacrolimus^d^ + CTX^d^ + Bari/6	0.06 2 mg, bid	PR/24
18	5/F	24.2	6/9,43/52,76/80	Refractory skin and muscle involvement	N/A	Negative	CS^a^ + MTX^d^ + IVIG^b^ + Tacrolimus^d^ + HCQ^c^ + CTX^d^ CS^a^ + MTX^d^ + Tacrolimus^d^ + HCQ^c^ + CTX^d^ + Bari/6	0.10 2 mg, qd	PR/4,PR/12, CR/24
19	2/F	35.5	4/9,40/52,61/80	Refractory skin and muscle involvement	Focal atrophy, perimysial and/or perivascular infiltration of mononuclear cells	Negative	CS^a^ + MTX^d^ + IVIG^d^ CS^a^ + MTX^d^ + IVIG^d^ + Bari/3 CS^a^ + MTX^d^ + IVIG^d^/3	0.25 2 mg, qm (0.5 month), 2 mg, bid (escalation)	CR/4
20	4/M	38.6	4/9,46/52,73/80	Refractory skin and muscle involvement	Focal atrophy, perimysial and/or perivascular infiltration of mononuclear cells	Negative	CS^a^ + MTX^d^ + IVIG^d^ + Tacrolimus^d^ CS^a^ + MTX^d^ + IVIG^d^ + Tacrolimus^d^ + Bari/3 CS^a^ + MTX^d^ + IVIG^d^ + Tacrolimus^d^/3	0.07 2 mg, qd	CR/4

^a^Decrease the dose after baricitinib initiation.

^b^Withdraw before baricitinib initiation.

^c^Withdraw after baricitinib initiation.

^d^The dose of the drug remains the same dose after baricitinib initiation.

^e^Additional or intensified use of other drugs after baricitinib initiation.

*Baricitinib was withdrawn because of disease relapse at 24 weeks. P, patient; F, female; M, male; CMAS, childhood myositis assessment scale; MMT, manual muscle testing; CR, complete responder; PR, partial responder; NR, non-responder; ILD, interstitial lung disease; CADM, clinically amyopathic dermatomyositis; CS, corticosteroid; MTX, methotrexate; MMF, mycophenolatemofetil; CTX, cyclophosphamide; CsA, cyclosporine A; IVIG, intravenous immunoglobulin; HCQ, hydroxychloroquine; INF, InfliximabI; N/A, not available.

### The effectiveness of baricitinib therapy

The skin rash improved in 9 patients (45%) at week 4, in 15 (75%) at week 12, and in 19 (95%) at week 24. Compared with the baseline, skin-DAS was significantly decreased at week 12 [2.0 (0, 3.0) vs. 6.0 (4.0, 7.3), *p* < 0.05] and week 24 [0 (0, 1.0) vs. 6.0 (4.0, 7.3), *p* < 0.05]. Compared with the baseline, the CMAS score was significantly increased at week 12 [46.0 (42.0, 52.0) vs. 41.0 (29.0, 44.0), *p* < 0.023] and week 24 [50.0 (45.0, 52.0) vs. 41.0 (29.0, 44.0), *p* < 0.006], as was the MMT-8 score at week 24 [79.0 (77.0, 80.0) vs. 73.0 (61.0, 76.0), *p* < 0.011].

**FIGURE 1:**
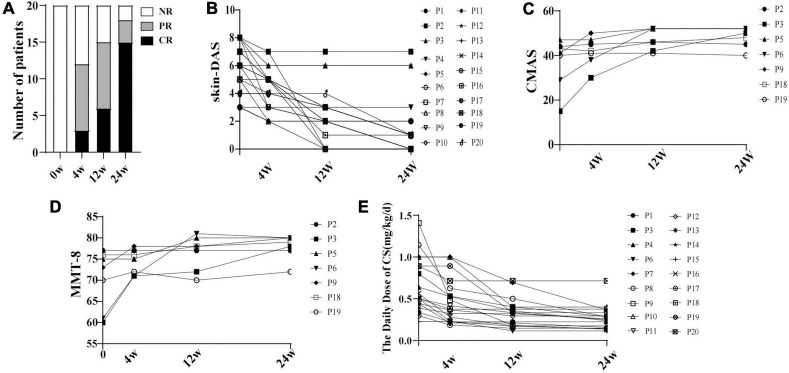
The disease activity changing in 20 patients with JDM with baricitinib treatment. **(A)** Proportion of the 20 patients with JDM achieving CR and/or PR within 24 weeks at different follow-up times with baricitinib treatment. **(B)** Decrease of skin-DAS of 20 patients. **(C)** Increase of CMAS of 7 patients. **(D)** Increase of MMT of 7 patients. **(E)** Decrease of the daily dose of CS. NR, non-response; CR, complete response; PR, partial response; DAS, disease activity score; p, patient; CMAS, childhood myositis assessment scale.

The CR and PR were achieved in 19 of 20 patients at 24 weeks after baricitinib therapy, including 15 (79%) with CR (Figure 1). The X-rays indicated that calcinosis was improved in one patient (P3) and stabilized in two (P2 and P12). ILD was improved in four patients (P4, P5, P9, and P17) and stabilized in one (P14). MAS was resolved in P9.

### Add-on therapy of baricitinib was helpful for tapering corticosteroids

Compared with the baseline, the daily CS dose was decreased by 37% at week 12 [0.33 (0.18, 0.40) vs. 0.53 (0.42, 1.0), *P* < 0.05], and by 49% at week 24 [0.27 (0.17, 0.37) vs. 0.53 (0.42, 1.0), *P* < 0.05] in patients who achieved CR and PR. Comparing to the week 4, the CS dosage was decreased significantly at week 24 [0.27 (0.17, 0.37) vs. 0.40 (0.23, 0.65), *P* < 0.007] (Figure 1).

### Safety of baricitinib therapy

There were 26 AEs in 10 patients (Table 3), most of which manifested as mild upper airway infections. The P13 had a slight increase in liver enzymes (ALT from 57 to 85 U/L) after taking the baricitinib without the addition of other medications, or with other muscle enzymes increasing, or clinical disease activity. The P1 experienced increased creatinine after baricitinib initiation (creatinine from 60 to 71 U/L), but not yet to the point of renal dysfunction. Hospitalization and temporary discontinuation of baricitinib were required in only one patient (P6, who had herpes zoster infection). There were no venous thrombosis, malignancy, and fatalities recorded in this study.

**TABLE 3:** Adverse events of baricitinib in the treatment of severe and/or refractory JDM (*n* = 20).

Adverse events- no (%)	
Upper respiratory infection	18(69%)
Fungus infection	2(7%)
Gastrointestinal disorder	2(7%)
Herpes zoster	1(4%)
Elevation of liver enzymes	1(4%)
Elevation of creatinine	1(4%)
Elevation of uric acid	1(4%)

There were 26 AEs in 10 patients.

## Discussion

Our study included the largest number of JDM cases treated with baricitinib to date. This study included 19 cases with a recurrent or refractory rashes that are dependent on CS therapy. Due to the expense and the lack of medical insurance, most patients in our study received doses of baricitinib that were lower than those in previous studies (17, 26). Our results showed skin rash was improved in 95% of the patients and all patients who had muscle weakness at the entry were improved at week 24. The skin-DAS and CMAS were significantly changed at week 12 and week 24, the MMT-8 was significantly improved at week 24. The CR and PR were achieved in 90% of the patients at week 24 after the baricitinb therapy.

Voyer et al. found that a new-onset JDM case with severe skin ulcerations improved to CR after 1.7 months of baricitinib initiation (15). Another previous study reported that skin rash was improved in four cases with refractory JDM after 4 weeks or later (17). Almost half of our cases were improved at week 4, while significant improvement in skin rash was noted at week 12 in skin-DAS (*P* < 0.05). These observations indicate that the baricitinib is clinically beneficial for skin lesions.

Our case series included seven patients with muscle involvement: six with mild muscle weakness and one (P3) with severe muscle weakness. The reason for a low proportion of muscle involvement may be the result of a long period of traditional treatment. In addition, P11 was diagnosed as clinically amyopathic dermatomyositis (CADM) based on Sontheimer criteria (27). A previous case report found that a JAK inhibitor was effective for muscle weakness in four patients who received baricitinib (15, 16, 28). We found significant differences in CMAS and MMT-8 scores after treatment. Our results indicated that baricitinib improved muscle weakness and P3 was markedly improved at 4 weeks after baricitinib treatment.

A previous study proposed that baricitinib may be helpful for tapering the CS dose. In some case reports, a JAK inhibitor may have even more beneficial when used alone (12). In this study, the daily median CS dosage decreased from 0.53 to 0.27 mg/kg/d (*P* < 0.05) after treatment in patients who got a response. The findings suggest that the baricitinib was effective, was fast acting, and was helpful in tapering CS dosage.

Calcinosis is a therapeutic challenge in JDM. There are previous case reports of calcinosis improving or stabilizing in four patients who received a JAK inhibitor, including two who were also treated with baricitinib (13, 15, 18). Our study found that two of three cases improved, while the third stabilized; baricitinib was effective for recurrent rash, and even calcinosis.

In recent years, some case reports have indicated that a JAK inhibitor is highly effective in treating JDM-associated ILD, with a combined effect of IFN signaling down-regulation and reduced expressions of proinflammatory cytokines (13, 16, 28–32). Five of our cases were complicated with ILD, stabilized (*n* = 2), improved (*n* = 2), or were resolved (*n* = 1) after baricitinib therapy, which manifested by HRCT or clinical improvement. While baricitinib might be an effective therapy for ILD, the number of cases was small and so further research is needed.

Interference with IFN *α/β* expression could control catastrophic hyper inflammation in MAS (33). There was no use of the baricitinib in patients with JDM with MAS, but given the successful application in hemophagocytic lymphohistiocytosis (HLH) (34, 35), we decided to apply it in JDM-related MAS. Comprehensive treatment that included IVMP, IVIG, tocilizumab, and baricitinib led to disease remission in one patient (P9) who experienced the cytokine storm, which suggests that baricitinib is an effective treatment for MAS in the real world.

Baricitinib was found to be well tolerated and safe in previous studies (18, 29). The most common AE in our study was a mild respiratory infection. One patient infected with herpes zoster had to temporarily suspend baricitinib use.

At the 24th week, P2 and P5 were classified as “NR,” the reason may be: P2 was a severe case with refractory skin ulcerations and calcinosis, however, because the patient did not have medical insurance, baricitinib was only used for 12 weeks, short duration of use may be the reason why this patient did not respond to it; P5 entered add-on treatment with baricitinib because of a persistent facial rash, but he was not covered by health insurance, so the patient agreed to receive a relatively small dose of baricitinib at 0.04 mg/kg/d.

Our study had some limitations. It was a single-center study with a short observation period, and it was carried out in a routine clinical practice situation, meaning that the effects of basic and combined medications may have influenced the observed effectiveness of the baricitinib. And as shown in Table 2, many of the patients used substandard dose and dosing intervals of baricitinib below the recommended dose based on weight and renal function (36). Besides, the lack of some critical clinical information did not allow us to examine the American College of Rheumatology/European League Against Rheumatism response criteria in JDM (37). We also did not measure the type I IFN levels and IFN response gene signatures of the patients.

## Conclusion

This study has indicated that baricitinib combined with CS and other immunosuppressants is effective and safe for refractory or severe patients with JDM, especially in recurrent skin rashes. Baricitinib was helpful for tapering the daily CS dose in patients. We also observed baricitinib to be effective in ILD, MAS, and calcinosis in some patients. However, a multicenter study with a longer observation period needs to be performed in the future.

## Data availability statement

The original contributions presented in this study are included in the article/Supplementary material, further inquiries can be directed to the corresponding author.

## Ethics statement

The studies involving human participants were reviewed and approved by the Ethics Committee of Children’s Hospital of Zhejiang University School of Medicine. Written informed consent to participate in this study was provided by the participants’ legal guardian/next of kin. Written informed consent was obtained from the individual(s), and minor(s)’ legal guardian/next of kin, for the publication of any potentially identifiable images or data included in this article.

## Author contributions

ZW was responsible for the collection of clinical information, statistical analyses, figures, data interpretation, and manuscript preparation. QZ was responsible for critical review of the statistical analyses and the manuscript. XSX, ZW, QZ, WX, ML, JW, LZ, YX, and XFX assisted in the clinical data collection. ML is the project leader of the study, involved in the conceptualization of the project, the study design, and preparation of the manuscript. All authors read and approved the final manuscript.
